# The Mediterranean as a melting pot: Phylogeography of *Loxosceles rufescens* (Sicariidae) in the Mediterranean Basin

**DOI:** 10.1371/journal.pone.0210093

**Published:** 2018-12-31

**Authors:** Marc Massa, Enric Planas, Carles Ribera

**Affiliations:** Departament de Biologia Evolutiva, Ecologia i Ciències Ambientals and Institut de Recerca de la Biodiversitat (IRBio), Facultat de Biologia, Universitat de Barcelona, Barcelona, Spain; National Cheng Kung University, TAIWAN

## Abstract

The species *Loxosceles rufescens* is native to the Mediterranean but considered cosmopolitan because it has been dispersed worldwide. A previous study revealed 11 evolutionary lineages across the Mediterranean, grouped into two main clades, without any clear phylogeographic pattern. The high genetic diversity within this species (p-distances of up to 7.8% in some Mediterranean lineages), together with the results obtained with different species delimitation methods (GMYC, TCS) could indicate the existence of cryptic species. Here we compare the mitochondrial and microsatellite diversity to elucidate if the lineages of *L*. *rufescens* in the Mediterranean should be considered different species (cryptic species) or populations of the same species. To do so, we analyzed the *cox1* diversity of 196 individuals, of which, we genotyped 148, sampled from 19 localities across the Mediterranean. STRUCTURE analyses of microsatellite data identified two genetic clusters of *L*. *rufescens*. One cluster included individuals from Western Mediterranean localities (Iberian Peninsula, Morocco, Balearic Islands) and Israel, while the second one grouped individuals from Italian and Greek localities, including Sardinia, Sicily and Tunisia. These patterns suggest that geographic proximity is the more significant factor in the clustering with microsatellite data and shows the existence of gene flow between the nearest geographic areas, even if the individuals belong to different mitochondrial lineages or clades. The lack of correspondence between both genetic markers suggests that the evolutionary lineages found within *L*. *rufescens* should not be considered different species. We conclude that these phylogenetic linages and their distribution may be the result of the maternal evolutionary history of the species and human-mediated dispersion.

## Introduction

Spiders of the genus *Loxosceles* Heineken and Lowe, 1832 (Araneae: Sicariidae) are widely known for their medical importance [1]. Their bite can cause a clinical condition known as loxoscelism, which is characterized by cutaneous necrosis in mammals [2]. Loxoscelism was not documented until the mid-twentieth century, and it has often been misdiagnosed [3] because its symptomatology is similar to other affections [4]. Even so, there are known cases across the entire distribution range of the genus, including the Mediterranean Basin, where *Loxosceles rufescens* (Dufour 1820) is widely distributed [1,3,5,6]. *Loxosceles rufescens* is endemic to the Mediterranean; however, it has now expanded its distribution due to human-mediated transportation, and it is considered cosmopolitan [7]. Because this species is associated with urban habitats the existence of loxoscelism cases becomes more plausible [8].

The Mediterranean Basin is considered an important biodiversity hotspot, and it is included among the main biodiversity hotspots for conservation priorities [9–10]. Multiple factors have promoted this biodiversity, for instance, the presence of multiple European glacial refugia and diverse geological events [6, 11–13].

The spider genus *Loxosceles* comprises 127 species that are predominantly distributed in the American continent (98 species) as well as in Africa (26 species) and Asia (3 species but see [14]) [7]. The high species diversity of the genus in the Americas contrasts with its low diversity in Africa, which is most likely due to the scarce taxonomic work that has been conducted in the last continent [5,15–18]. Duncan et al. [19] suggested that the diversity of this genus in Northwestern Africa could be underestimated given the greater diversity found in other regions such as South America or North America. This diversity is starting to be recognized, and recently, six new species of *Loxosceles* from the Canary Islands were described as members of the *L*. *rufescens* group [8,20]. Given the simple morphological genitalia that characterize *Loxosceles* species, genetic approaches can be very useful for delimiting species [8,19].

Its ability to expand into new territories is enhanced by its high synanthropism [3,6,21] and resistance to starvation [3,22]. In the regions where *L*. *rufescens* has been introduced, it can be found inhabiting infrastructures and areas related to human activities [3,22]. Similar to other *Loxosceles* species, it has a low dispersal capacity because, unlike other spiders, it does not disperse by ballooning [3,14]. *L*. *rufescens* individuals tend to be sedentary and show a strong preference to stay in the same location for long periods of time or their entire life [2,3].

Studying the genetic diversity of *L*. *rufescens* in the Mediterranean Basin Planas et al. [6] proposed the existence of eleven mitochondrial lineages based on an analysis of the first subunit of cytochrome c oxidase (*cox1*). They also point out that these 11 lineages could be considered as putative species based on the results obtained with different methods of species delimitation (GMYC, TCS). The lineages were grouped into two clades: A and B. The first clade is a combination of six lineages (A1 to A6), and the second clade is composed of five (B1 to B5). However, the lineages were adjusted to two completely different phylogeographic patterns: while some lineages from Morocco and the Iberian Peninsula were well structured, the remaining lineages were distributed all over the Mediterranean without a definite geographical pattern. The authors [6] proposed that the current genetic diversity probably originated in allopatry and was promoted by successive glaciations during the Pleistocene and that protracted human activities impacted the current distributional patterns of *L*. *rufescens* within the Mediterranean Basin and mixed the geographic structuration of the lineages. The same authors indicated that the uncovered phylogeographic patterns should be strengthened with additional nuclear data.

In this contribution, we compare the mtDNA and the microsatellite diversity within *L*. *rufescens* to elucidate if the mitochondrial lineages of *L*. *rufescens* that exist in the Mediterranean should be considered different species (cryptic species) or if these are populations of the same species. To do so, we sampled numerous populations of *L*. *rufescens* throughout the Mediterranean by considering the geographical distribution and the representation of the mitochondrial lineages.

If the mitochondrial lineages must be considered different species, we would expect to find a correspondence between the mitochondrial and microsatellite clusters, which would suggest that alleles of individuals from different lineages or clades would remain distinctive after secondary contact. In contrast, if the microsatellite information does not match with the outlook provided by mitochondrial data, the microsatellite loci would delimitate geographically structured genetic clusters whose individuals could belong to different mitochondrial lineages. Since alleles would be able to mix, we expect that the allelic frequencies would show more similarity between nearer populations than distant ones and favoring the hypothesis that the current genetic variability would have originated in several glacial refugia during the Pleistocene and that their posterior mixture around the Mediterranean Basin would have been facilitated by human-mediated dispersion.

## Materials and methods

### Phylogenetic analysis

Our dataset includes a total of 196 terminals (S1 Table), 128 *cox1* sequences obtained in Planas et al. [6], and (following the same procedures) 68 new sequences. This allowed us to identify the lineages of individuals that previously lacked a mitochondrial sequence. The *cox1* partial sequences were aligned in GENEIOUS v6.1.6 [23] using ClustalW [24] with default parameters. We partitioned the data by the codon position and simultaneously explored the best partitioning schemes and substitution models for RAxML using PartitionFinder v1.0.1 [25] under a Bayesian information criterion.

To infer the mitochondrial lineage of each individual, a maximum likelihood (ML) analysis was performed using RAx-ML 7.0.3 [26] with the graphical front-end RAxMLGUI 1.3 [27]. We applied an ML plus thorough bootstrap in one run with 1000 replicates by applying the GTRG model. Position of the root of the tree was estimated implicitly in BEAST v.1.7.4 [28] and used for rooting RAxML trees as in Planas et al. [6].

### The dataset of microsatellites

**Fig 1:**
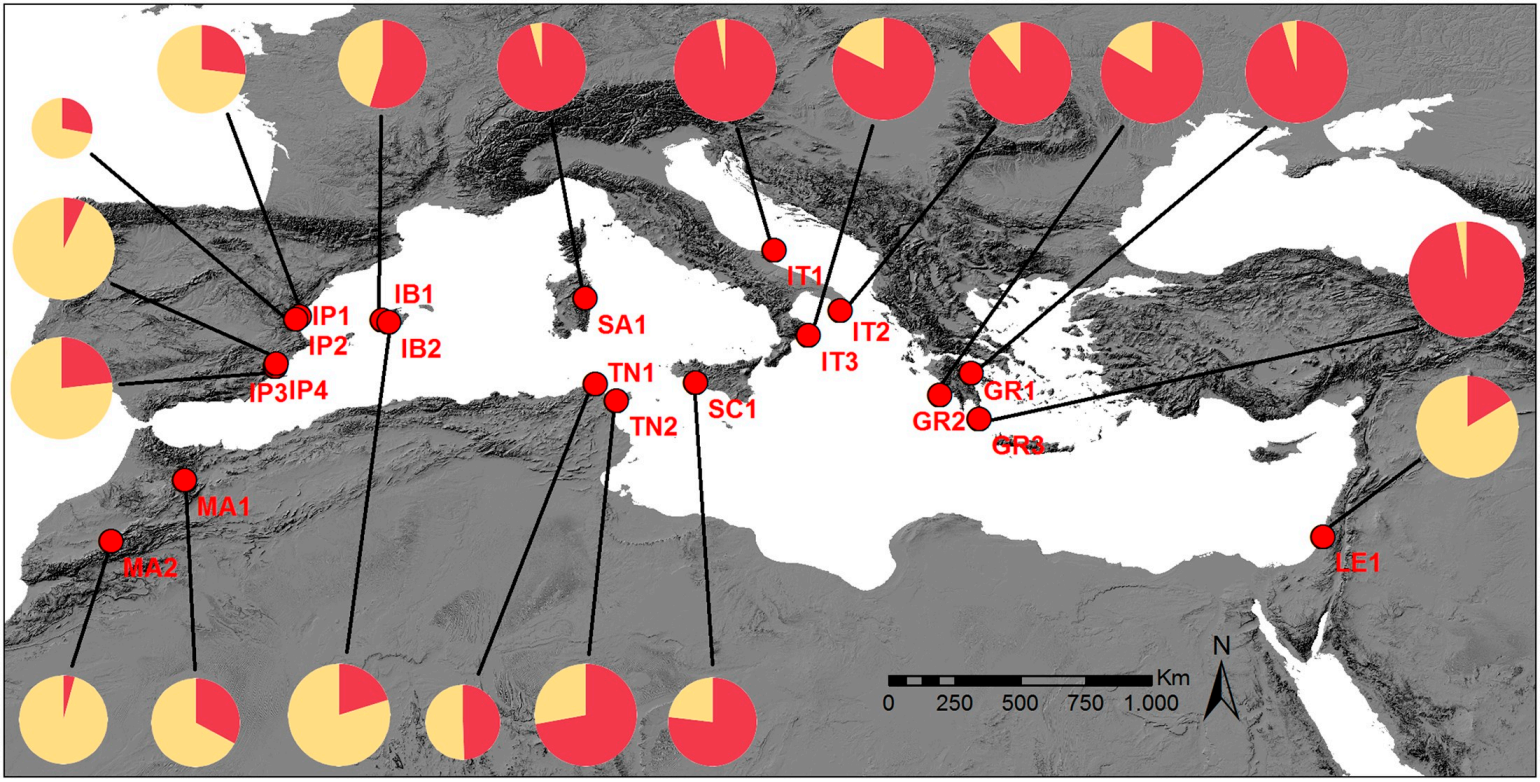
Sampling of *Loxosceles rufescens* throughout the Mediterranean basin. Circles represent the proportional assignation of microsatellite loci to each genetic cluster. Abbreviations: GR (Greece), IB (Balearic Islands), IP (Iberian Peninsula), IT (Italy), LE (Israel), MA (Morocco), SA (Sardinia), SC (Sicily), TN (Tunisia).

We genotyped 148 individuals sampled from 19 localities across the Mediterranean Basin (Fig 1) for seven microsatellite loci developed specifically for *L*. *rufescens* [29]. We conducted PCR reactions in a final volume 10 μl using Biotools Pfu DNA Polymerase (Biotools). The PCR conditions were 94 °C for 1 min followed by 35 cycles of 94 °C for 30 s; the annealing temperatures ranged between 42 °C and 66 °C according to the microsatellite loci for 20 to 35 s, followed by 72 °C for 30 s and a final extension at 72 °C for 3 min. We pooled the PCR products according to dye type and expected allele range to genotype them in an ABI 3730XLs automated sequencer at Macrogen (Seoul) with an internal size standard of 500 LIZ. For allele scoring, we used Microsatellite Plugin 1.4 in GENEIOUS v6.1.6 [23]. We calculated the missing data percentages for each individual, population, locality and mitochondrial c*ox1* linage in R [30] using custom scripts that are available upon request.

To identify the genetic clusters, we used microsatellite data with a method based on Bayesian clustering, which is implemented in the program STRUCTURE [31]. With this methodology, we detected genetic clusters that minimize departures from Hardy-Weinberg equilibrium using allelic frequencies [31]. We conducted the analyses by considering the admixture model and uncorrelated allele frequencies among the populations and performing 20 independent runs with 100000 iterations and 50000 burn-ins for each K between K = 1 and K = 19 (the total number of localities). We inferred the most likely number of clusters using Evanno’s method [32] implemented in Structure Harvester [33]. We combined the twenty independent results for the most likely K-value with the software CLUMPP [34] and this was graphically represented with Distruct [35]. We arranged the localities based on the longitudinal position (from west to east).

To evaluate the effect of the missing data in the previous analysis, we tested five additional subsets of the most complete dataset. First (subset 1), we removed individuals lacking information for two out of seven microsatellites loci. In subset 2, we removed all the individuals with missing data. In subset 3, we removed the microsatellite locus with the highest value of missing data. In subset 4, we removed the locus with the highest value of missing data and individuals without information for two of the remaining six loci. Finally, in subset 5, we again removed the locus with the highest value of missing data and all the individuals with missing data.

## Results and discussion

### Cox 1 Phylogenetic analysis

**Fig 2:**
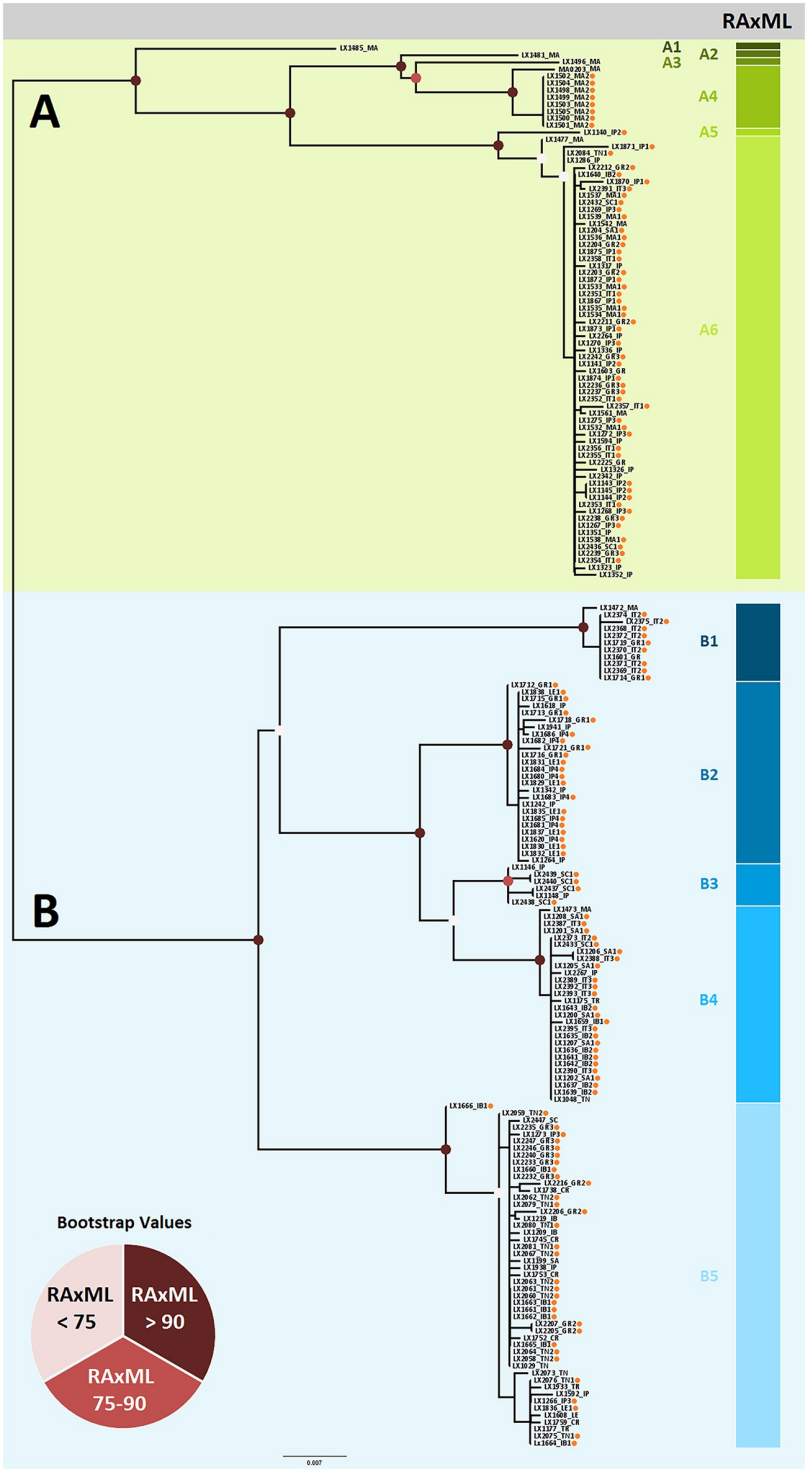
Maximum likelihood tree based on the *cox1* sequences. Node circles represent the bootstrap values as shown in the legend. The clade A is indicated by green, and the clade B by blue. Colored bars on the right indicates the different lineages from each clade. Spots on the terminals indicates individuals with microsatellites information. Abbreviations: CR (Crete), GR (Greece), IB (Balearic Islands), IP (Iberian Peninsula), IT (Italy), LE (Israel), MA (Morocco), SA (Sardinia), SC (Sicily), TN (Tunisia), TR (Turkey). The position of the root was estimated implicitly in BEAST v.1.7.4 and used for rooting RAxML trees.

The *cox1* alignment included a total of 196 sequences, 148 of which came from individuals that were also used in the microsatellite’s analyses. The phylogenetic ML tree (Fig 2) shows the same topology reported by Planas et al. [6] grouping the individuals of *L*. *rufescens* in two main clades: A and B. The bootstrap values for each node were comparable to the values obtained in Planas et al. [6].

The geographic distribution of the haplotypes is comparable to Planas et al. [6]. It is noteworthy that in Planas et al. [6] the lineage B3 included exclusive representatives from the Iberian Peninsula, whereas in our dataset and by including new data, it also contained Sicilian representatives (SC1). This finding suggests that lineages found in a restricted number of localities could be detected in more localities with thorough sampling. Thus, even lineages distributed in a few close localities could have a wider geographic distribution.

### The dataset of microsatellites

We obtained the genotype of seven microsatellite loci for 148 individuals of *L*. *rufescens*. Six individuals were dismissed because of their excessively high values of missing data. We obtained a total of 56 alleles across the seven loci, and the number of alleles per locus ranged between five in the loci ME031 and ME067 and 12 in the locus ME012. This dataset includes individuals with information of at least five of the seven loci and shows 6.44% of the missing data. The number of alleles per locus and the values of missing data per locus are found in S2 Table. The values of missing data per locality range between 0% in IB2 and TN1 and 12.5% in LE1 (S3 Table), and the values of missing data per linage range between 3.57% in B3 and 28.57% in A5 (S4 Table). The missing data values per loci range between 0% in ME113 and 16.2% in ME031.

The missing data for the five additional datasets amounted to 5.12% for subset 1, 4.81% for subset 3 (ME031 removed), 4.2% for subset 4 and 0% for subsets 2 and 5. The number of alleles in subset 1 was reduced to 54, that in subsets 2, 3 and 4 was reduced to 51, and in subset 5, the number was reduced to 48. The number of alleles per locus ranged between five in ME067 and ME031 and 11 in ME012 in subset 1, between five in ME067 and 11 in ME067 in subsets 2 and 5, and between five in ME067 and 12 in ME012 in the subsets 3 and 4.

### Genetic clusters

**Fig 3:**
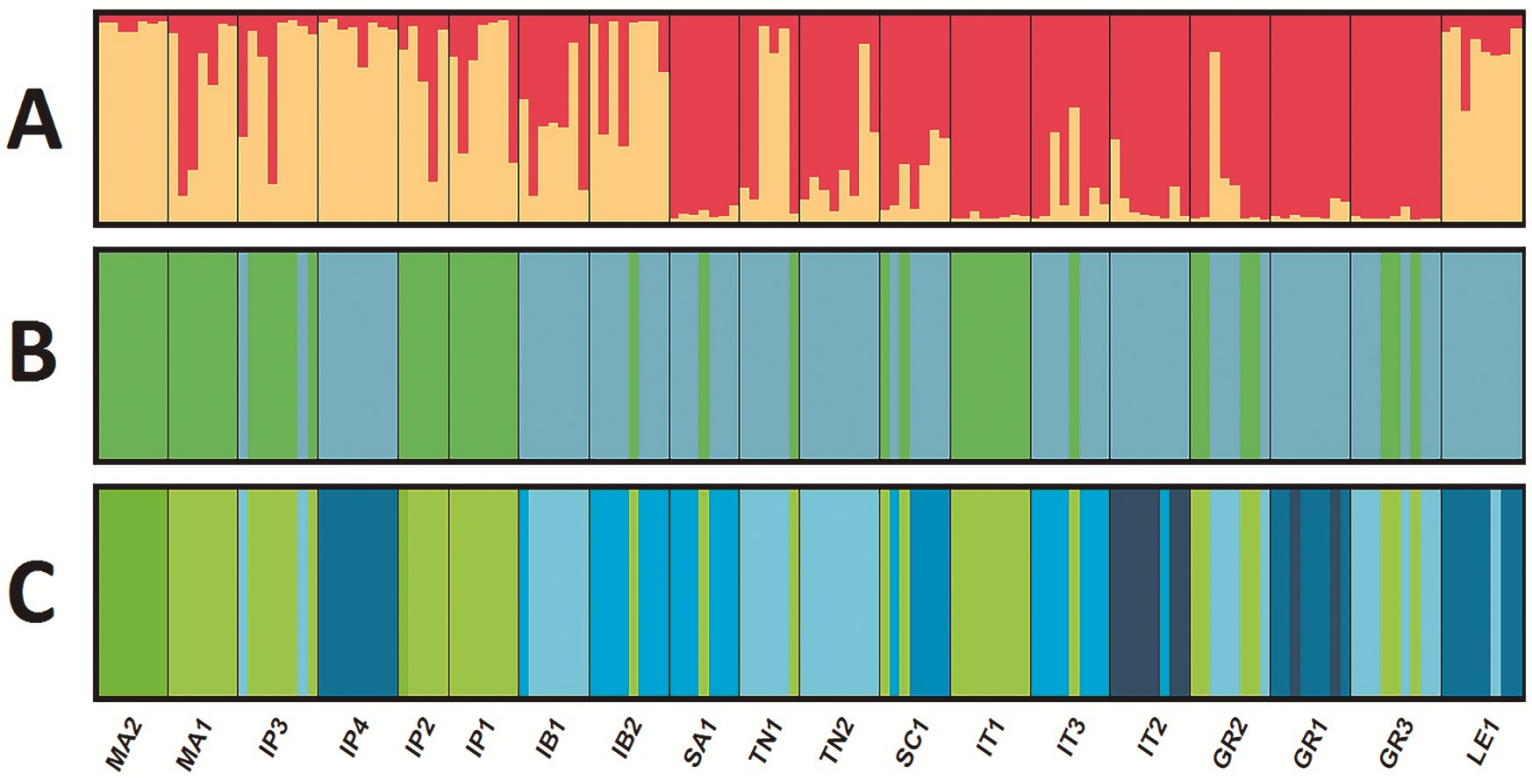
A: STRUCTURE results based on microsatellite loci for K = 2. Verticals lines represent proportional assignation to each genetic cluster. Black lines delimit the different localities; yellow—cluster 1, red—cluster 2. B: Representation of the phylogenetic clades based on the *cox1* sequences to which each individual belongs; green—clade A, blue—clade B. C: Representation of the lineages to which each individual belongs; Same colors for the lineages as colored bars in Fig 2. Abbreviations: GR (Greece), IB (Balearic Islands), IP (Iberian Peninsula), IT (Italy), LE (Israel), MA (Morocco), SA (Sardinia), SC (Sicily), TN (Tunisia).

We obtained K = 2 as the optimal number of clusters in the STRUCTURE analysis following Evanno’s method (Fig 3). The majority of the individuals of the Iberian localities (IP1, IP2, IP3 and IP4), Morocco (MA2), Balearic Islands (IB2) and Israel (LE1) were grouped into cluster 1, whereas most individuals of the Greek (GR1, GR2 and GR3) and Italian (IT1, IT2 and IT3) localities, including Sardinia and Sicily (SA1 and SC1) and Tunisia (TN2), were grouped into cluster 2. Individuals from TN1, IB1 and MA1 were assigned to both clusters (1 and 2). It should be noted that 13 of the 142 analyzed individuals show an assignation value close to 50% and thus have an indefinite affiliation.

These results show moderate geographical structuration of the genetic clusters obtained based on the microsatellite information (Figs 1 and 3). Cluster 1 includes roughly the individuals of the western localities (Morocco and Iberian Peninsula), and cluster 2 includes the individuals of the eastern localities (Italy and Greece). However, individuals from the easternmost locality, LE1, were assigned to cluster 1. We interpret this fact as a consequence of lower sampling in North Africa and the Eastern Mediterranean; therefore, more thorough samplings could better define the range of both genetic clusters.

The results obtained with subsets 1 to subset 5 did not greatly differ from the results obtained with the complete dataset. The assignment value of individuals closest to 50% only changed in some datasets (S1 Fig), which supported that missing data are not substantially affecting our results.

### Must all mitochondrial lineages be considered the same species?

In this study, we observed a general incongruity between the microsatellite clusters and the mitochondrial lineage, that is, not all the individuals that belong to one mitochondrial lineage correspond to the same microsatellite clusters as inferred by STRUCTURE (Fig 3). In both microsatellite clusters, there are individuals of the same mitochondrial lineage (Fig 3). For example, while the individuals from localities IB2 and SA1 were assigned to different microsatellite clusters, most of them belong to the mitochondrial lineage (i.e., B4). Similarly, individuals from the three Italian localities (IT1, IT2 and IT3) and assigned to the same microsatellite cluster belong to different mitochondrial lineages (i.e., A6, B1 and B4).

We observed a general geographic pattern by considering the microsatellite clusters. As noted above, one genetic cluster is compounded by the individuals from the western Mediterranean localities (Morocco and Iberian Peninsula) and others by individuals from Italy and Greece. This pattern suggests that geographic proximity is a more significant factor in the clustering with microsatellite data. This is despite the fact that in some cases, the assignation of the individuals is not markedly homogeneous for the individuals of the same locality, for example, TN1 (n = 6), where half of the individuals were assigned to cluster 1 and the other half to cluster 2. However, the west-east geographic pattern obtained by microsatellites is broken by the assignation of the easternmost individuals (LE1) to the western cluster. This fact is probably a consequence of scarce sampling in North Africa and the Eastern Mediterranean, or a consequence of human-mediated dispersal, since 7 of the 8 individuals analyzed belong to clade B2, where most of them are from Iberian Peninsula. If we exclude this exception, the results show a clear west-east geographic pattern.

Overall, our results suggest that neither the mitochondrial lineages nor the clades should be considered different species. Our dataset included several localities (IP2, IP3, IB1, IB2, SA1, TN1, SC1, IT2, IT3, GR1, GR2, GR3 and LE1) with individuals belonging to different mitochondrial clades or lineages. Since the individuals were clustered together with the microsatellite data and thus were not structured according to the mitochondrial lineages or clades, the hypothesis of the existence of cryptic species is not supported. Several localities (i.e., IP4, LE1 and GR1) included individuals of a single mitochondrial lineage, (i.e., lineage B2) and were assigned to different microsatellite clusters, again not supporting the hypothesis of the existence of different cryptic species.

Planas et al. [6] proposed that the different mitochondrial lineages originated in several glacial refugia by divergence during the Pleistocene. The same authors also explained the complex phylogeographic pattern and the lack of strong geographic structuration in *L*. *rufescens* as a consequence of external factors and mostly the influence of human-mediated dispersal. These external factors would have altered (and they probably would still be altering) the phylogeographic structuration of this species, which allows populations from different Mediterranean regions to resume interaction. This hypothesis is supported by the results obtained by microsatellite data, which show the existence of gene flow between the nearest geographic areas even if the individuals belong to different mitochondrial clades. Therefore, the different mitochondrial linages could have been mixed up in the Mediterranean Basin.

## Conclusions

In the present study, we analyzed multiple microsatellite loci from different localities of *L*. *rufescens* around the Mediterranean Basin. Using STRUCTURE, we detected the presence of two different genetic clusters. These two clusters are approximately geographically structured in a way such that the individuals from Morocco and the Iberian Peninsula would be differentiated from representatives of Italy and Greece.

The mitochondrial lineages and clades inferred from *cox1* do not correspond to those obtained from microsatellites clusters. Our interpretation of the lack of correspondence between the two genetic markers is that the different evolutionary lineages found within *L*. *rufescens* should not be considered different species. The different lineages originated in distinct glacial refugia during the Pleistocene and were distributed through human-mediated dispersion. However, the employment of a higher number of microsatellites and more localities with larger sample sizes would be necessary to be able to conclusively determine the existence of these genetic clusters and resolve how the populations of *L*. *rufescens* are structured in the Mediterranean Basin. Additionally, the analysis of other nuclear genetic markers would be beneficial to increase our knowledge regarding the evolutionary history of this species.

## Supporting information

S1 Fig STRUCTURE results based on the microsatellite loci for K = 2 with all the individuals. 2: STRUCTURE results based on the microsatellite loci for K = 2 without individuals with more than one loci missing. 3: STRUCTURE results based on the microsatellite loci for K = 2 without individuals with missing loci. A. Including all the microsatellite loci. B. Excluding microsatellite locus ME031. Abbreviations: GR (Greece), IB (Balearic Islands), IP (Iberian Peninsula), IT (Italy), LE (Israel), MA (Morocco), SA (Sardinia), SC (Sicily), TN (Tunisia).
(PDF)

S1 Table Locality (geographic coordinates), mitochondrial DNA lineage and GenBank accession number for the 196 individuals included in the *cox1* phylogenetic analyses. In bold, individuals genotyped for the seven microsatellite loci Sequences with * are new for this study. Abbreviations: GR (Greece), IB (Balearic Islands), IP (Iberian Peninsula), IT (Italy), LE (Israel), MA (Morocco), SA (Sardinia), SC (Sicily), TN (Tunisia).
(DOCX)

S2 Table Number of alleles and missing data per locus. (DOCX)

S3 Table Number of individuals and missing data per locality. Abbreviations: GR (Greece), IB (Balearic Islands), IP (Iberian Peninsula), IT (Italy), LE (Israel), MA (Morocco), SA (Sardinia), SC (Sicily), TN (Tunisia).
(DOCX)

S4 Table Number of individuals and missing data per lineage. (DOCX)
