# Supplementary figures and images for: The Mediterranean as a melting pot: Phylogeography of Loxosceles rufescens (Sicariidae) in the Mediterranean Basin
Source: PLoS One. 2018 Dec 31;13(12):e0210093. doi: 10.1371/journal.pone.0210093 (PMC6312272; doi:10.1371/journal.pone.0210093)

1)

A

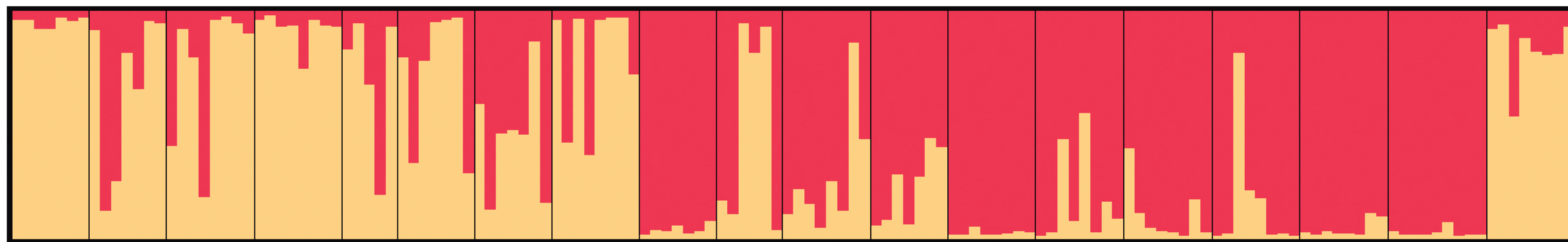

B

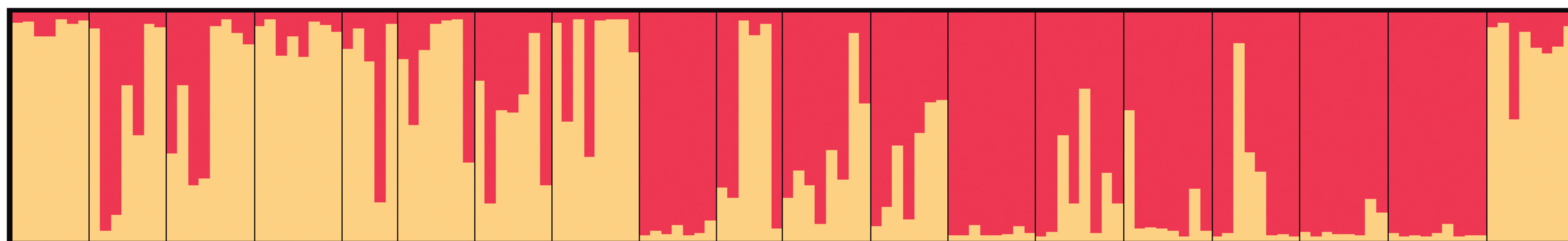

2)

A

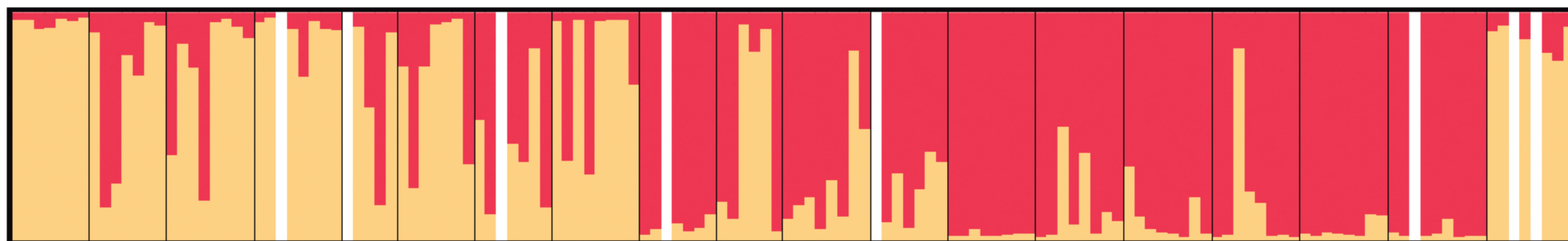

B

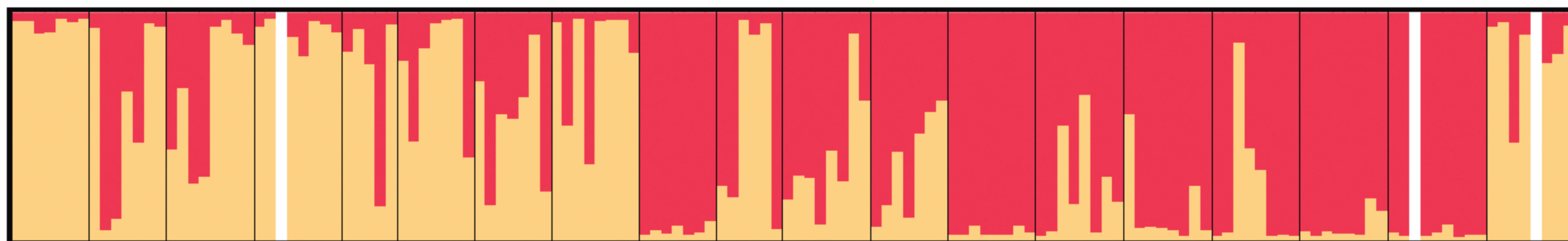

3)

A

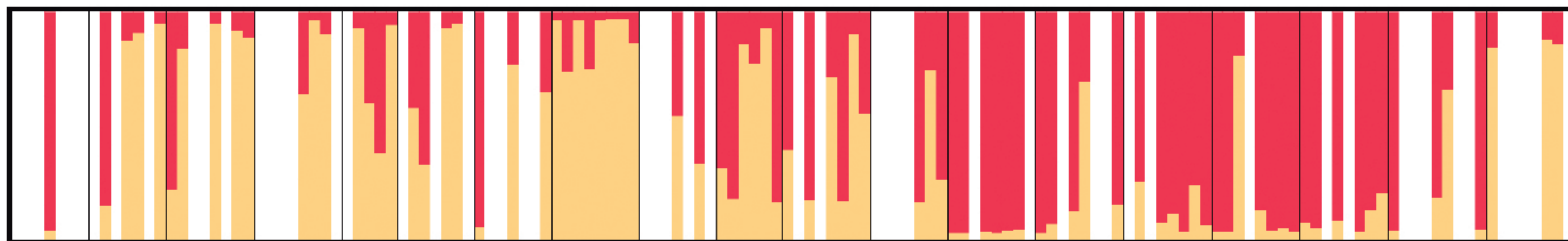

B

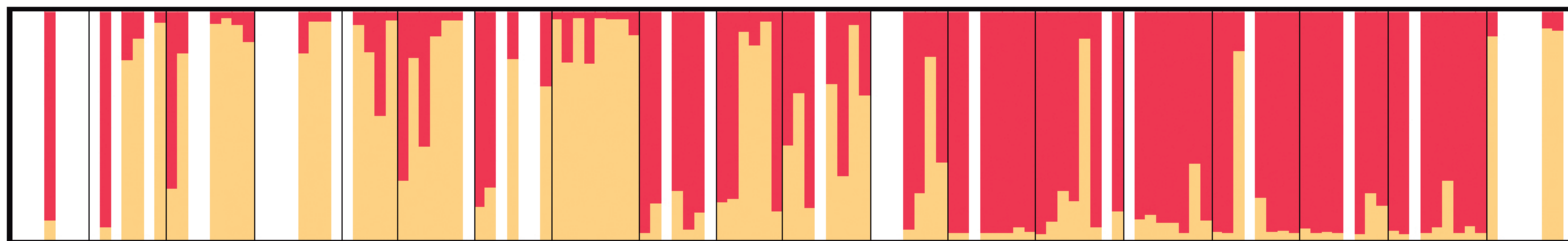

MA2 MA1 IP3 IP4 IP2 IP1 IB1 IB2 SA1 TN1 TN2 SC1 IT1 IT3 IT2 GR2 GR1 GR3 LE1

Supplement: S1 Fig — 2: STRUCTURE results based on the microsatellite loci for K = 2 without individuals with more than one loci missing. 3: STRUCTURE results based on the microsatellite loci for K = 2 without individuals with missing loci. A. Including all the microsatellite loci. B. Excluding microsatellite locus ME031. Abbreviations: GR (Greece), IB (Balearic Islands), IP (Iberian Peninsula), IT (Italy), LE (Israel), MA (Morocco), SA (Sardinia), SC (Sicily), TN (Tunisia). (PDF) [file pone.0210093.s001.pdf]
