# Supplementary material for: The Mediterranean as a melting pot: Phylogeography of Loxosceles rufescens (Sicariidae) in the Mediterranean Basin
Source: PLoS One. 2018 Dec 31;13(12):e0210093. doi: 10.1371/journal.pone.0210093 (PMC6312272; doi:10.1371/journal.pone.0210093)
Supplement: S1 Table — Abbreviations: GR (Greece), IB (Balearic Islands), IP (Iberian Peninsula), IT (Italy), LE (Israel), MA (Morocco), SA (Sardinia), SC (Sicily), TN (Tunisia). (DOCX) [file pone.0210093.s002.docx]

| Individual Code | Region | Longitude | Latitude | Lineage | Accession number (Cox1) |
| --- | --- | --- | --- | --- | --- |
| **LX1712** | GR | 22.7517 | 37.7224666667 | B2 | KJ560724 |
| **LX1713** | GR | 22.7517 | 37.7224666667 | B2 | KJ560725 |
| **LX1714** | GR | 22.7517 | 37.7224666667 | B1 | KJ560726 |
| **LX1715** | GR | 22.7517 | 37.7224666667 | B2 | KJ560727 |
| **LX1716** | GR | 22.7517 | 37.7224666667 | B2 | KJ560728 |
| **LX1718** | GR | 22.7517 | 37.7224666667 | B2 | MH198698^*^ |
| **LX1719** | GR | 22.7517 | 37.7224666667 | B1 | MH198699^*^ |
| **LX1721** | GR | 22.7517 | 37.7224666667 | B2 | MH198700^*^ |
| **LX2203** | GR | 21.65729 | 36.960084 | A6 | MH198716^*^ |
| **LX2204** | GR | 21.65729 | 36.960084 | A6 | MH198717^*^ |
| **LX2205** | GR | 21.65729 | 36.960084 | B5 | MH198718^*^ |
| **LX2206** | GR | 21.65729 | 36.960084 | B5 | MH198719^*^ |
| **LX2207** | GR | 21.65729 | 36.960084 | B5 | MH198720^*^ |
| **LX2211** | GR | 21.65729 | 36.960084 | A6 | KJ560796 |
| **LX2212** | GR | 21.65729 | 36.960084 | A6 | KJ560797 |
| **LX2216** | GR | 21.65729 | 36.960084 | B5 | KJ560798 |
| **LX2232** | GR | 23.018313 | 36.14744 | B5 | KJ560804 |
| **LX2233** | GR | 23.018313 | 36.14744 | B5 | KJ560805 |
| **LX2235** | GR | 23.018313 | 36.14744 | B5 | KJ560806 |
| **LX2236** | GR | 23.018313 | 36.14744 | A6 | KJ560807 |
| **LX2237** | GR | 23.018313 | 36.14744 | A6 | KJ560808 |
| **LX2238** | GR | 23.018313 | 36.14744 | A6 | KJ560809 |
| **LX2239** | GR | 23.018313 | 36.14744 | A6 | KJ560810 |
| **LX2240** | GR | 23.018313 | 36.14744 | B5 | KJ560811 |
| **LX2242** | GR | 23.018313 | 36.14744 | A6 | KJ560813 |
| **LX2246** | GR | 23.018313 | 36.14744 | B5 | KJ560814 |
| **LX2247** | GR | 23.018313 | 36.14744 | B5 | KJ560815 |
| **LX1659** | IB | 2.5335333333 | 39.5333 | B4 | KJ560711 |
| **LX1660** | IB | 2.5335333333 | 39.5333 | B5 | KJ560712 |
| **LX1661** | IB | 2.5335333333 | 39.5333 | B5 | MH198692^*^ |
| **LX1662** | IB | 2.5335333333 | 39.5333 | B5 | MH198693^*^ |
| **LX1663** | IB | 2.5335333333 | 39.5333 | B5 | MH198694^*^ |
| **LX1664** | IB | 2.5335333333 | 39.5333 | B5 | MH198695^*^ |
| **LX1665** | IB | 2.5335333333 | 39.5333 | B5 | MH198696^*^ |
| **LX1666** | IB | 2.5335333333 | 39.5333 | B5 | MH198697^*^ |
| **LX1635** | IB | 2.76845 | 39.4582333333 | B4 | KJ560699 |
| **LX1636** | IB | 2.76845 | 39.4582333333 | B4 | KJ560700 |
| **LX1637** | IB | 2.76845 | 39.4582333333 | B4 | KJ560701 |
| **LX1639** | IB | 2.76845 | 39.4582333333 | B4 | KJ560703 |
| **LX1640** | IB | 2.76845 | 39.4582333333 | A6 | KJ560704 |
| **LX1641** | IB | 2.76845 | 39.4582333333 | B4 | KJ560705 |
| **LX1642** | IB | 2.76845 | 39.4582333333 | B4 | KJ560706 |
| **LX1643** | IB | 2.76845 | 39.4582333333 | B4 | KJ560707 |
| **LX1874** | IP | -0.2886833333 | 39.6678666667 | A6 | KJ560774 |
| **LX1867** | IP | -0.2886833333 | 39.6678666667 | A6 | MH198701^*^ |
| **LX1870** | IP | -0.2886833333 | 39.6678666667 | A6 | MH198702^*^ |
| **LX1871** | IP | -0.2886833333 | 39.6678666667 | A6 | MH198703^*^ |
| **LX1872** | IP | -0.2886833333 | 39.6678666667 | A6 | MH198704^*^ |
| **LX1873** | IP | -0.2886833333 | 39.6678666667 | A6 | MH198705^*^ |
| **LX1875** | IP | -0.2886833333 | 39.6678666667 | A6 | MH198706^*^ |
| **LX1140** | IP | -0.41551 | 39.549464 | A5 | KJ560569 |
| **LX1141** | IP | -0.41551 | 39.549464 | A6 | KJ560570 |
| **LX1143** | IP | -0.41551 | 39.549464 | A6 | KJ560572 |
| **LX1144** | IP | -0.41551 | 39.549464 | A6 | KJ560573 |
| **LX1145** | IP | -0.41551 | 39.549464 | A6 | KJ560574 |
| **LX1266** | IP | -1.13036 | 37.92965 | B5 | KJ560603 |
| **LX1267** | IP | -1.13036 | 37.92965 | A6 | KJ560604 |
| **LX1268** | IP | -1.13036 | 37.92965 | A6 | KJ560605 |
| **LX1269** | IP | -1.13036 | 37.92965 | A6 | MH198679^*^ |
| **LX1270** | IP | -1.13036 | 37.92965 | A6 | KJ560606 |
| **LX1272** | IP | -1.13036 | 37.92965 | A6 | KJ560607 |
| **LX1273** | IP | -1.13036 | 37.92965 | B5 | KJ560608 |
| **LX1275** | IP | -1.13036 | 37.92965 | A6 | KJ560609 |
| **LX1620** | IP | -1.09445 | 38.0362 | B2 | KJ560696 |
| **LX1680** | IP | -1.09445 | 38.0362 | B2 | KJ560717 |
| **LX1681** | IP | -1.09445 | 38.0362 | B2 | KJ560718 |
| **LX1682** | IP | -1.09445 | 38.0362 | B2 | KJ560719 |
| **LX1683** | IP | -1.09445 | 38.0362 | B2 | KJ560720 |
| **LX1684** | IP | -1.09445 | 38.0362 | B2 | KJ560721 |
| **LX1685** | IP | -1.09445 | 38.0362 | B2 | KJ560722 |
| **LX1686** | IP | -1.09445 | 38.0362 | B2 | KJ560723 |
| **LX2351** | IT | 15.98725 | 41.94056 | A6 | MH198721^*^ |
| **LX2352** | IT | 15.98725 | 41.94056 | A6 | MH198722^*^ |
| **LX2353** | IT | 15.98725 | 41.94056 | A6 | MH198723^*^ |
| **LX2354** | IT | 15.98725 | 41.94056 | A6 | KJ560834 |
| **LX2355** | IT | 15.98725 | 41.94056 | A6 | KJ560835 |
| **LX2356** | IT | 15.98725 | 41.94056 | A6 | KJ560836 |
| **LX2357** | IT | 15.98725 | 41.94056 | A6 | MH198724^*^ |
| **LX2358** | IT | 15.98725 | 41.94056 | A6 | MH198725^*^ |
| **LX2368** | IT | 18.24284 | 39.86921 | B1 | MH198726^*^ |
| **LX2369** | IT | 18.24284 | 39.86921 | B1 | KJ560837 |
| **LX2370** | IT | 18.24284 | 39.86921 | B1 | KJ560838 |
| **LX2371** | IT | 18.24284 | 39.86921 | B1 | KJ560839 |
| **LX2372** | IT | 18.24284 | 39.86921 | B1 | MH198727^*^ |
| **LX2373** | IT | 18.24284 | 39.86921 | B4 | MH198728^*^ |
| **LX2374** | IT | 18.24284 | 39.86921 | B1 | MH198729^*^ |
| **LX2375** | IT | 18.24284 | 39.86921 | B1 | MH198730^*^ |
| **LX2387** | IT | 17.16621 | 39.01587 | B4 | MH198731^*^ |
| **LX2388** | IT | 17.16621 | 39.01587 | B4 | MH198732^*^ |
| **LX2389** | IT | 17.16621 | 39.01587 | B4 | MH198733^*^ |
| **LX2390** | IT | 17.16621 | 39.01587 | B4 | KJ560843 |
| **LX2391** | IT | 17.16621 | 39.01587 | A6 | KJ560844 |
| **LX2392** | IT | 17.16621 | 39.01587 | B4 | MH198734^*^ |
| **LX2393** | IT | 17.16621 | 39.01587 | B4 | MH198735^*^ |
| **LX2395** | IT | 17.16621 | 39.01587 | B4 | KJ560845 |
| **LX1829** | LE | 34.799977 | 32.116533 | B2 | KJ560762 |
| **LX1830** | LE | 34.799977 | 32.116533 | B2 | KJ560763 |
| **LX1831** | LE | 34.799977 | 32.116533 | B2 | KJ560764 |
| **LX1832** | LE | 34.799977 | 32.116533 | B2 | KJ560765 |
| **LX1835** | LE | 34.799977 | 32.116533 | B2 | KJ560768 |
| **LX1836** | LE | 34.799977 | 32.116533 | B5 | KJ560769 |
| **LX1837** | LE | 34.799977 | 32.116533 | B2 | KJ560770 |
| **LX1838** | LE | 34.799977 | 32.116533 | B2 | KJ560771 |
| **LX1532** | MA | -4.23691 | 34.0573 | A6 | KJ560661 |
| **LX1538** | MA | -4.23691 | 34.0573 | A6 | KJ560662 |
| **LX1533** | MA | -4.23691 | 34.0573 | A6 | MH198686^*^ |
| **LX1534** | MA | -4.23691 | 34.0573 | A6 | MH198687^*^ |
| **LX1535** | MA | -4.23691 | 34.0573 | A6 | MH198688^*^ |
| **LX1536** | MA | -4.23691 | 34.0573 | A6 | MH198689^*^ |
| **LX1537** | MA | -4.23691 | 34.0573 | A6 | MH198690^*^ |
| **LX1539** | MA | -4.23691 | 34.0573 | A6 | MH198691^*^ |
| **LX1498** | MA | -6.76811 | 31.95973 | A4 | KJ560654 |
| **LX1499** | MA | -6.76811 | 31.95973 | A4 | KJ560655 |
| **LX1500** | MA | -6.76811 | 31.95973 | A4 | MH198680^*^ |
| **LX1501** | MA | -6.76811 | 31.95973 | A4 | MH198681^*^ |
| **LX1502** | MA | -6.76811 | 31.95973 | A4 | MH198682^*^ |
| **LX1503** | MA | -6.76811 | 31.95973 | A4 | MH198683^*^ |
| **LX1504** | MA | -6.76811 | 31.95973 | A4 | MH198684^*^ |
| **LX1505** | MA | -6.76811 | 31.95973 | A4 | MH198685^*^ |
| **LX1200** | SA | 9.50725 | 40.2886111111 | B4 | KJ560588 |
| **LX1201** | SA | 9.50725 | 40.2886111111 | B4 | MH198674^*^ |
| **LX1202** | SA | 9.50725 | 40.2886111111 | B4 | KJ560589 |
| **LX1204** | SA | 9.50725 | 40.2886111111 | A6 | MH198675^*^ |
| **LX1205** | SA | 9.50725 | 40.2886111111 | B4 | MH198676^*^ |
| **LX1206** | SA | 9.50725 | 40.2886111111 | B4 | MH198677^*^ |
| **LX1207** | SA | 9.50725 | 40.2886111111 | B4 | KJ560590 |
| **LX1208** | SA | 9.50725 | 40.2886111111 | B4 | MH198678^*^ |
| **LX2432** | SC | 13.29294 | 37.39064 | A6 | MH198736^*^ |
| **LX2433** | SC | 13.29294 | 37.39064 | B4 | MH198737^*^ |
| **LX2436** | SC | 13.29294 | 37.39064 | A6 | KJ560854 |
| **LX2437** | SC | 13.29294 | 37.39064 | B3 | MH198738^*^ |
| **LX2438** | SC | 13.29294 | 37.39064 | B3 | MH198739^*^ |
| **LX2439** | SC | 13.29294 | 37.39064 | B3 | MH198740^*^ |
| **LX2440** | SC | 13.29294 | 37.39064 | B3 | MH198741^*^ |
| **LX2081** | TN | 9.84616 | 37.33186 | B5 | KJ560787 |
| **LX2084** | TN | 9.84616 | 37.33186 | A6 | KJ560788 |
| **LX2075** | TN | 9.84616 | 37.33186 | B5 | MH198712^*^ |
| **LX2076** | TN | 9.84616 | 37.33186 | B5 | MH198713^*^ |
| **LX2079** | TN | 9.84616 | 37.33186 | B5 | MH198714^*^ |
| **LX2080** | TN | 9.84616 | 37.33186 | B5 | MH198715^*^ |
| **LX2058** | TN | 10.58439 | 36.77661 | B5 | KJ560783 |
| **LX2059** | TN | 10.58439 | 36.77661 | B5 | KJ560784 |
| **LX2060** | TN | 10.58439 | 36.77661 | B5 | MH198707^*^ |
| **LX2061** | TN | 10.58439 | 36.77661 | B5 | MH198708^*^ |
| **LX2062** | TN | 10.58439 | 36.77661 | B5 | MH198709^*^ |
| **LX2063** | TN | 10.58439 | 36.77661 | B5 | MH198710^*^ |
| **LX2064** | TN | 10.58439 | 36.77661 | B5 | MH198711^*^ |
| **LX2067** | TN | 10.58439 | 36.77661 | B5 | KJ560785 |
| LX1485 | MA | 31.15739 | -9.69767 | A1 | KJ560651 |
| LX1481 | MA | 31.50096 | -9.6158 | A2 | KJ560650 |
| LX1496 | MA | 31.18955 | -8.05766 | A3 | KJ560653 |
| MA0203 | MA | 31.250088 | -7.983352 | A4 | GQ279225 |
| LX1477 | MA | 31.60901 | -9.65915 | A6 | KJ560649 |
| LX1286 | IP | 37.21327 | -1.82724 | A6 | KJ560613 |
| LX1542 | MA | 34.82454 | -2.08165 | A6 | KJ560663 |
| LX1317 | IP | 36.84788 | -2.0249 | A6 | KJ560623 |
| LX2264 | IP | 41.38605 | 2.1639 | A6 | KJ560819 |
| LX1336 | IP | 36.80214 | -2.14298 | A6 | KJ560632 |
| LX1603 | GR | 38.4523167 | 22.4213833 | A6 | KJ560686 |
| LX1561 | MA | 34.80333 | -2.39686 | A6 | KJ560665 |
| LX1594 | IP | 37.4763427 | -4.2792721 | A6 | KJ560680 |
| LX2225 | GR | 35.881197 | 23.305596 | A6 | KJ560801 |
| LX1326 | IP | 36.84535 | -2.01746 | A6 | KJ560627 |
| LX2342 | IP | 40.86461 | 0.501121 | A6 | KJ560830 |
| LX1351 | IP | 39.68481 | -0.30005 | A6 | KF717003 |
| LX1323 | IP | 36.84788 | -2.0249 | A6 | KJ560625 |
| LX1352 | IP | 39.68481 | -0.30005 | A6 | KJ560635 |
| LX1472 | MA | 32.50405 | -9.25307 | B1 | KJ560647 |
| LX1601 | GR | 39.1923333 | 23.9233333 | B1 | KJ560684 |
| LX1618 | IP | 38.0362 | -1.09445 | B2 | KJ560695 |
| LX1941 | IP | 36.44859 | -5.88949 | B2 | KJ560781 |
| LX1342 | IP | 36.80214 | -2.14298 | B2 | KJ560633 |
| LX1242 | IP | 38.0362 | -1.09445 | B2 | KJ560600 |
| LX1264 | IP | 38.0362 | -1.09445 | B2 | KJ560602 |
| LX1146 | IP | 37.383717 | -4.786571 | B3 | KJ560575 |
| LX1148 | IP | 39.536261 | -0.6263104 | B3 | KJ560576 |
| LX1473 | MA | 31.9564 | -9.31604 | B4 | KJ560648 |
| LX2267 | IP | 36.38845 | -5.65146 | B4 | KJ560820 |
| LX1175 | TR | 36.938501 | 31.169117 | B4 | KJ560580 |
| LX1048 | TN | 35.9165506 | 9.55883798 | B4 | KJ560562 |
| LX2447 | SC | 38.08555 | 12.67299 | B5 | KJ560855 |
| LX1738 | CR | 35.3508167 | 24.35395 | B5 | KJ560735 |
| LX1219 | IB | 39.609295 | 2.5991 | B5 | KJ560596 |
| LX1209 | IB | 39.3468472 | 3.18578889 | B5 | KJ560591 |
| LX1745 | CR | 35.2018667 | 24.16235 | B5 | KJ560738 |
| LX1199 | SA | 39.5305 | 9.59586111 | B5 | KJ560587 |
| LX1938 | IP | 37.61826 | -7.2 | B5 | KJ560779 |
| LX1753 | CR | 35.51075 | 24.06835 | B5 | KJ560744 |
| LX1752 | CR | 35.51075 | 24.06835 | B5 | KJ560743 |
| LX1029 | TN | 36.5101533 | 9.13655918 | B5 | KJ560561 |
| LX2073 | TN | 37.329 | 9.84616 | B5 | KJ560786 |
| LX1933 | TR | 36.777248 | 31.475971 | B5 | KJ560776 |
| LX1592 | IP | 37.99675 | -6.3422 | B5 | KJ560679 |
| LX1608 | LE | 30.897868 | 34.894342 | B5 | KJ560691 |
| LX1759 | CR | 35.3273167 | 23.5537833 | B5 | KJ560745 |
| LX1177 | TR | 36.9716667 | 31.5333333 | B5 | KJ560582 |
