# Supplementary material for: The Mediterranean as a melting pot: Phylogeography of Loxosceles rufescens (Sicariidae) in the Mediterranean Basin
Source: PLoS One. 2018 Dec 31;13(12):e0210093. doi: 10.1371/journal.pone.0210093 (PMC6312272; doi:10.1371/journal.pone.0210093)
Supplement: S2 Table — (DOCX) [file pone.0210093.s003.docx]

| Locus name | ME031 | ME103 | ME034 | ME067 | ME113 | ME088 | ME012 |
| --- | --- | --- | --- | --- | --- | --- | --- |
| Nº alleles | 5 | 11 | 8 | 5 | 9 | 6 | 12 |
| Percentage *missing data* | 16.20 | 8.45 | 8.45 | 1.41 | 0 | 8.45 | 2.11 |
