# Supplementary material for: The Mediterranean as a melting pot: Phylogeography of Loxosceles rufescens (Sicariidae) in the Mediterranean Basin
Source: PLoS One. 2018 Dec 31;13(12):e0210093. doi: 10.1371/journal.pone.0210093 (PMC6312272; doi:10.1371/journal.pone.0210093)
Supplement: S3 Table — Abbreviations: GR (Greece), IB (Balearic Islands), IP (Iberian Peninsula), IT (Italy), LE (Israel), MA (Morocco), SA (Sardinia), SC (Sicily), TN (Tunisia). (DOCX) [file pone.0210093.s004.docx]

| Locality | Number of individuals | Percentage of missing data |
| --- | --- | --- |
| MA2 | 7 | 12.24 |
| MA1 | 7 | 6.12 |
| IP3 | 8 | 5.36 |
| IP4 | 8 | 10.71 |
| IP2 | 5 | 5.71 |
| IP1 | 7 | 6.12 |
| IB1 | 7 | 10.20 |
| IB2 | 8 | 0 |
| SA1 | 7 | 12.24 |
| TN1 | 6 | 0 |
| TN2 | 8 | 3.57 |
| SC1 | 7 | 10.20 |
| IT1 | 8 | 3.57 |
| IT3 | 8 | 5.36 |
| IT2 | 8 | 3.57 |
| GR2 | 8 | 1.79 |
| GR1 | 8 | 3.57 |
| GR3 | 9 | 9.52 |
| LE1 | 8 | 12.50 |
