# Supplementary material for: The Mediterranean as a melting pot: Phylogeography of Loxosceles rufescens (Sicariidae) in the Mediterranean Basin
Source: PLoS One. 2018 Dec 31;13(12):e0210093. doi: 10.1371/journal.pone.0210093 (PMC6312272; doi:10.1371/journal.pone.0210093)
Supplement: S4 Table — (DOCX) [file pone.0210093.s005.docx]

| Lineage | A4 | A5 | A6 | B1 | B2 | B3 | B4 | B5 |
| --- | --- | --- | --- | --- | --- | --- | --- | --- |
| Nº individuals | 7 | 1 | 45 | 9 | 21 | 4 | 23 | 32 |
| Percentage *missing data* | 12.24 | 28.57 | 5.08 | 4.76 | 9.52 | 3.57 | 6.21 | 5.36 |
